# An Assessment of the Effectiveness of High Definition Cameras as Remote Monitoring Tools for Dolphin Ecology Studies

**DOI:** 10.1371/journal.pone.0126165

**Published:** 2015-05-12

**Authors:** Estênio Guimarães Paiva, Chandra Salgado-Kent, Marthe Monique Gagnon, Iain Parnum, Robert McCauley

**Affiliations:** 1 Centre for Marine Science and Technology, Curtin University, Perth, Western Australia, Australia; 2 Department of Environment and Agriculture, Curtin University, Perth, Western Australia, Australia; Sonoma State University, UNITED STATES

## Abstract

Research involving marine mammals often requires costly field programs. This paper assessed whether the benefits of using cameras outweighs the implications of having personnel performing marine mammal detection in the field. The efficacy of video and still cameras to detect Indo-Pacific bottlenose dolphins (*Tursiops aduncus*) in the Fremantle Harbour (Western Australia) was evaluated, with consideration on how environmental conditions affect detectability. The cameras were set on a tower in the Fremantle Port channel and videos were perused at 1.75 times the normal speed. Images from the cameras were used to estimate position of dolphins at the water’s surface. Dolphin detections ranged from 5.6 m to 463.3 m for the video camera, and from 10.8 m to 347.8 m for the still camera. Detection range showed to be satisfactory when compared to distances at which dolphins would be detected by field observers. The relative effect of environmental conditions on detectability was considered by fitting a Generalised Estimation Equations (GEEs) model with Beaufort, level of glare and their interactions as predictors and a temporal auto-correlation structure. The best fit model indicated level of glare had an effect, with more intense periods of glare corresponding to lower occurrences of observed dolphins. However this effect was not large (-0.264) and the parameter estimate was associated with a large standard error (0.113). The limited field of view was the main restraint in that cameras can be only applied to detections of animals observed rather than counts of individuals. However, the use of cameras was effective for long term monitoring of occurrence of dolphins, outweighing the costs and reducing the health and safety risks to field personal. This study showed that cameras could be effectively implemented onshore for research such as studying changes in habitat use in response to development and construction activities.

## Introduction

Research on non-captive marine mammals is often challenging and logistically onerous because the animals are generally difficult to access and detect due to their remoteness, mobility, and mostly elusive behaviour (e.g. diving for long periods) [1–3]. Field researchers are required to undertake long field programs that are costly and time consuming. Methods most commonly used to collect scientific information on marine mammals involve undertaking visual surveys from land, vessel, or aircraft either with the naked eye or using visual aids such as binoculars. The use of remote cameras in place of observers in the field is of increasing interest since this would decrease the health and safety risks to personel undertaking field work in the marine environment, and has the potential for collecting large data sets that can be post-processed with computational aids. High definition photographic and video cameras can also be used in conjunction with other non-intrusive techniques for use in quantitative and behavioural cetacean studies, such as passive acoustics [2, 4, 5] and theodolite tracking [6, 7].

Cameras have a long history of being used to measure ecological parameters in the terrestrial environment [8, 9]. Many of these studies have involved highly-mobile taxa such as birds [10, 11], mammals [12, 13], reptiles and amphibians [14]. Camera trapping, for instance, allows detection of species that are considered hard to record because they are rare or human-shy [9, 15]. This method consists of capturing images or videos of wildlife from a motion sensor or infrared activated camera, allowing data collection for animal abundance [16] and population studies [17], including those of species that move long distances and live in small groups. When considering a long-term approach, camera trapping is an effective tool for surveying mammals in the wild [18] which can contribute to more effective management actions.

Some examples of ecological and conservation planning applications using cameras include abundance and density estimation, species biodiversity, and anthropogenic impacts on fauna. Costs for fauna density estimates may be reduced by applying camera trap methods in which individual identification of animals is not necessary [19]. Trapping rate can be associated with density of terrestrial mammals based on average group size, day range, distance and angle within which the camera detected animals [19]. Using cameras during fishing activities is an efficient way to record and identify marine fauna presence, and to evaluate the fishing impacts over threatened and protected species [20]. Cameras also allow precise and complete measurements of animal behaviour as images of all the activities displayed by all animals within the camera’s field of view are recorded and can be reviewed multiple times and in slow motion [8, 21]. Given the onerous and costly nature of observing marine mammals over long periods in the field [22, 23], new techniques such as long-term implementation of cameras may provide a innovative approach for studying parameters such as the social structure and group composition [24] of marine mammals, as well as occurrence and occupancy. Images of cetaceans obtained from boats can be employed to calculate the range to animals at sea from the angle subtended between the horizon and the waterline of the target [25]. The use of technologies such as cameras also allows for the collection and storage of data for continued or future studies that are outside the scope of the initial study [7; 26].

Few studies, however, have tested the efficacy of different technologies, including the implementation of cameras, for marine mammal field studies [21] and compared their costs with standard field observations. This lack of information creates difficulty in standardizing methodologies and reducing the uncertainties in the selection of optimal methods. High definition cameras have methodological limitations which must be addressed if they are to be used to study free-ranging marine mammals. These limitations include high initial equipment costs [17, 24] and camera accuracy, which can be limited by biases associated with the field of view, general environmental conditions and location. Furthermore, the development of research technologies such as cameras for marine fauna is still in its early stages. Some techniques used for terrestrial fauna are not logistically practical for studying marine mammals [18]. The use of cameras to obtain information about the distribution of wildlife, while common in terrestrial contexts, has limitations when applied to small cetaceans. For example, land-based cameras can be used only in areas where the animals come close to the shore [24]. Video surveys are not commonly used because of the high capacity required for digital storage [8], however recent advances in information technology offer new opportunities for the use of video and photo recording which can potentially provide long-term data [21].

Thus, the current research aimed to better understand the advantages and limitations of using video and still cameras to further identify optimum methodologies to detect marine mammal presence, abundance, and behaviour. This project was conducted with Indo-pacific bottlenose dolphins (*Tursiops aduncus*) as a focal species and included the implementation of a still camera and a high definition video camera. The objectives of this research were: (a) to assess and compare the detection range of dolphins using still and video cameras; (b) to compare the performance of still and high definition video cameras in overall detection of the presence of dolphins; (c) to verify how environmental conditions affect detectability using video and still cameras; and (d) to assess the cost effectiveness of the use of cameras compared to field personnel to detect presence of dolphins. In this regard, this paper assesses whether the benefits of using cameras outweigh the costs and implications of having trained personnel recording observations in the field. This is an unusual approach for detecting marine mammals in the wild over long periods and, if considered practical and cost-effective, would also have applications for research questions, such as coastal marine mammal behaviour in response to anthropogenic disturbance.

## Methods

### Ethics statement

Observations of dolphins using cameras mounted on a jetty did not require approval by the Ethics and Safety Committee of Curtin University at the time the data were collected (2010), and does not require state permits for “taking” animals since the camera is remotely placed (and on a jetty) and does not cause disturbance to the animals. Data were collected within the Fremantle Inner Harbour, as part of work done for the Fremantle Port Authority during the Fremantle Inner Harbour Deepening Project. The methodology consisted of recording occurrence of Indo-Pacific bottlenose dolphins using high definition video and still cameras.

### Study site

**Fig 1:**
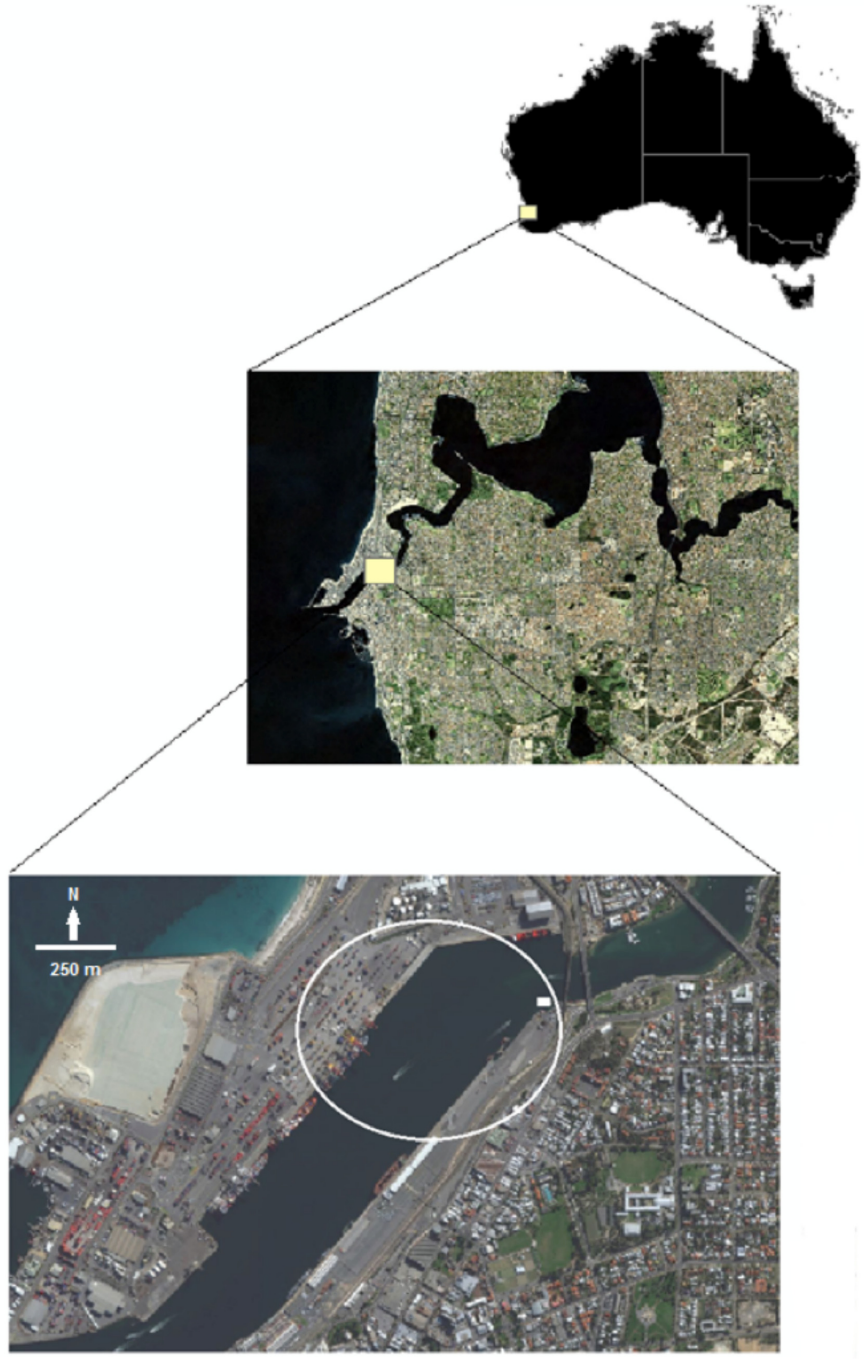
Location of the study site within the Fremantle Inner Harbour, Western Australia. The circle indicates the study area and the white box indicates the place where cameras were located. Source: ESRI ArcGlobe 10.0.

The study site was located within the Fremantle Inner Harbour (32.04°S 115.75°E), in the southwest region of Western Australia, 23 km south-west of the capital city of Perth. The Fremantle Inner Harbour (Fig 1) is located at the entrance of the Fremantle Port, which is the main location for import and export activities within Western Australia. The most easterly area of the Fremantle Inner Harbour is also the mouth of the Swan River system which is inhabited by several species of fish which a resident community of Indo-Pacific bottlenose dophins prey upon [27]. Dolphins have been documented to swim upstream and downstream of the channel on a regular basis [28, 29].

### High-definition video and still camera setup

**Fig 2:**
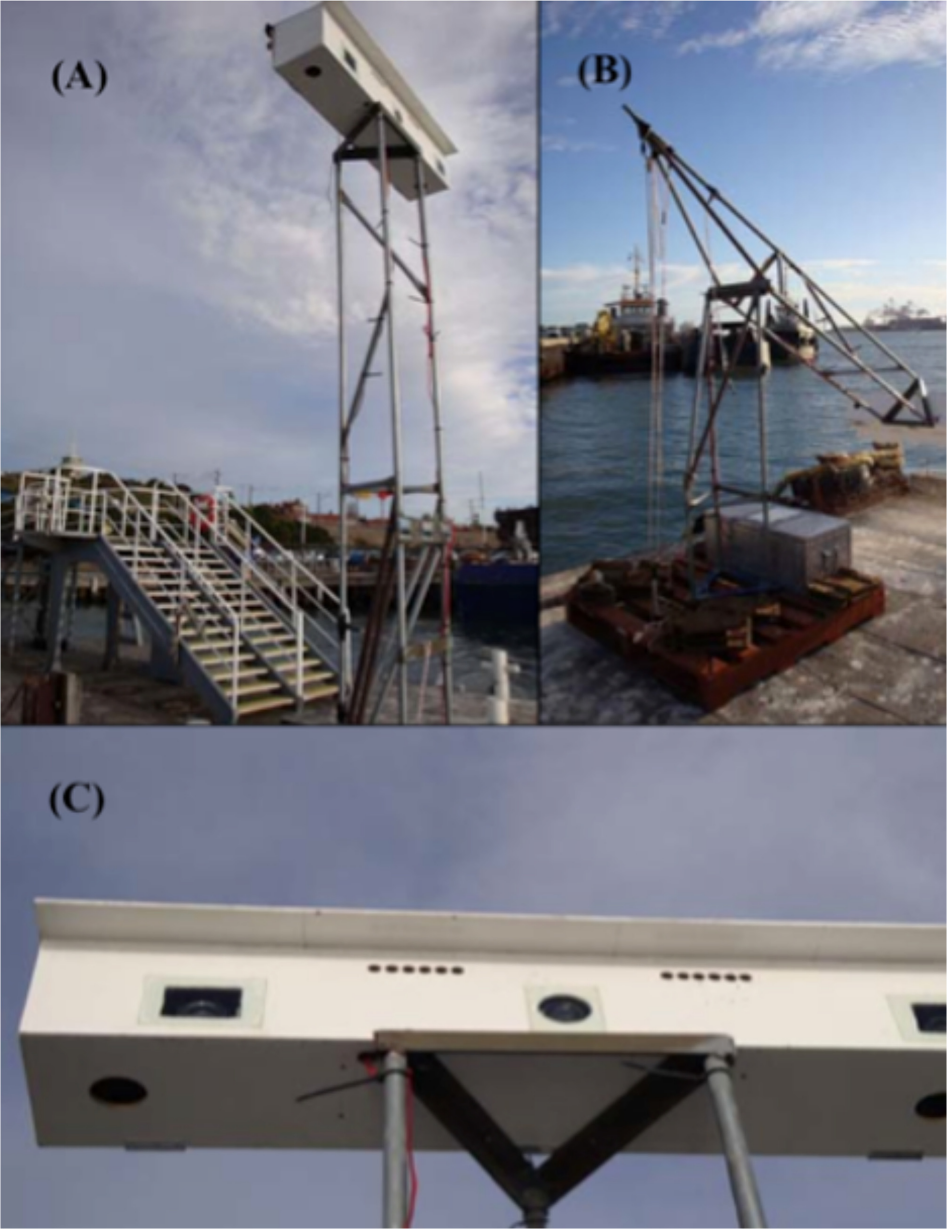
Equipment. Camera box mounted at the top of the camera tower (A); camera tower lowered for equipmemt servicing (B); camera box showing central window of the video camera and left window used for the still camera (the right window was for an additional camera not used in this study) (C).

One high definition video camera was strategically set at the top of a purpose-built 3-meter tower, which was placed at the end of the Fremantle Port small craft jetty on the west side of the Fremantle Inner Harbour, between the 16^th^ of April 2010 and the 2^nd^ of August 2010. The camera was a high-definition Sony HDR-XR520V (1440 x 1020 pixels) powered by two car batteries which required changing every 72 hours. The camera was zoomed out completely and focused at infinity to have the widest field of view, and was linked to a timer and set to record between 06:00 and 18:00 daily. The digital still camera was mounted aside the video camera. The still camera was a Canon PowerShot SX1 IS (3840 x 2160 pixels) set to Custom mode, manual focus, infinity zoom and safety manual focus off. This camera was also linked to the timer which was set to trigger the camera’s shutter release once every 20 seconds over the period betwen 06:00 and 18:00. The digital still camera recorded to 16Gb SD memory cards, which were capable of storing photos taken over the period of four 12-hour days before downloading was required. The cameras were mounted inside a water-tight box with windows placed at the locations of the camera lenses (Fig 2).

### High-definition video camera and still camera data acquisition

In order to determine the best playback speed in which to analyze the videos, a “trial” analysis was performed by four trained observers who watched the same six videos each at four different speeds (normal, 1.75 times faster than the normal speed, 2 times and 4 times faster than normal), and recorded the number of groups of dolphins detected. The speeds specified here were those available in VLC Media Player Version 2.1.5 Rincewind [30] for viewing video images. The videos were strategically chosen to include a range of environmental conditions, including rain, glare, sea states and light level conditions. The results were then compared to quantify dolphin misdetections as a function of video speed.

Misdetections ocurred when videos were watched at speeds greater than 1.75. Furthermore, some analysts viewing video footage at speeds greater than two times the normal speed reported feeling dizzy when watching the videos for extended periods due to constant changes and movements in the images. As a result, 1.75 times faster than the normal speed was selected as the maximum, effective speed for viewing videos.

For the purpose of comparing cameras’ detection ranges and overall effectiveness, 42 days worth of videos from the high-definition camera and images from the still camera were perused. Videos were perused at 1.75 times the normal speed until dolphins were detected. When dolphins were detected the video was then played at normal speed, and the following information collected: the time dolphins were observed at (UTC + 8:00), number of dolphins in each group observed, and the total time that the group stayed within the field of view of the camera.

For the number of dolphins within each group, the minimum, maximum and the best estimate were recorded. The best estimate represents a point estimate determined by the observer, according to the conditions at the time, taking into account the possibility of double counts [31]. The term “transit event” was used here to define the time an individual or a group of dolphins was first detected within the field of view to the time it left the field of view of the camera. Given the influence that weather conditions can have on detectability of dolphins during field observations, qualitative measures of environmental data were collected, including sea state, rain, glare, haziness, light and cloud cover. Sea state was classified according to the Beaufort scale (1 to 12) and rain was recorded as “present” or “absent”. The amount of glare was classified using a scale from 0 to 4, in which “0” represented “no glare” and “4” severe glare (when most of the field of view was covered by surface glare) [32]. Haziness was recorded as 0 for “no haze” or, if it was present, it was relative to the far side of the harbor (with “front” equal to haze level 1, “middle” to haze level 2 and “far” to level 3) and the degree of visibility was recorded based on the area covered. The amount of light was recorded as 1 (dawn and dusk) and 2 (day). The light from lamp posts on the wharf reflected on the water, creating intercalated visible sections during periods of low sunlight. Cloud cover was quantified by dividing the visible sky into 8 equal parts and then expressed as a percentage. Even though the shape was not a circle in the sky divided by 8 pie-shaped partitions as commonly done by personnel working in the field, this method provided a similar, practical percentage cover estimate approach by splitting the visible sky in the field of view of the camera into 8 rectangular partitions. Finally, the presence of sunspots and droplets on the camera lens was recorded by dividing the field of view where the inner harbor water surface was visible into four sections and then rating the percentage of the total obscured by sunspots or droplets according to the sections filled.

Data on numbers of dolphins, group size, transit events, and environmental conditions was extracted first from the videos recorded. Photographs from the still camera taken during periods in which dolphins were detected on the video camera were then analyzed to identify misdetections by the still camera. The aim was to compare the performance of still and high definition video cameras in overall detection of the presence of dolphins. An analysis of all still photographs that coincided with the periods of analysed video was not undertaken due to the huge amount of time that would have been required for manual perusal of still photos. The approach for analysing still photographs consisted of manually searching for dolphins in photographs taken 5 minutes prior to, to 5 minutes after, the first detection of the individual or group in the video.

**Fig 3:**
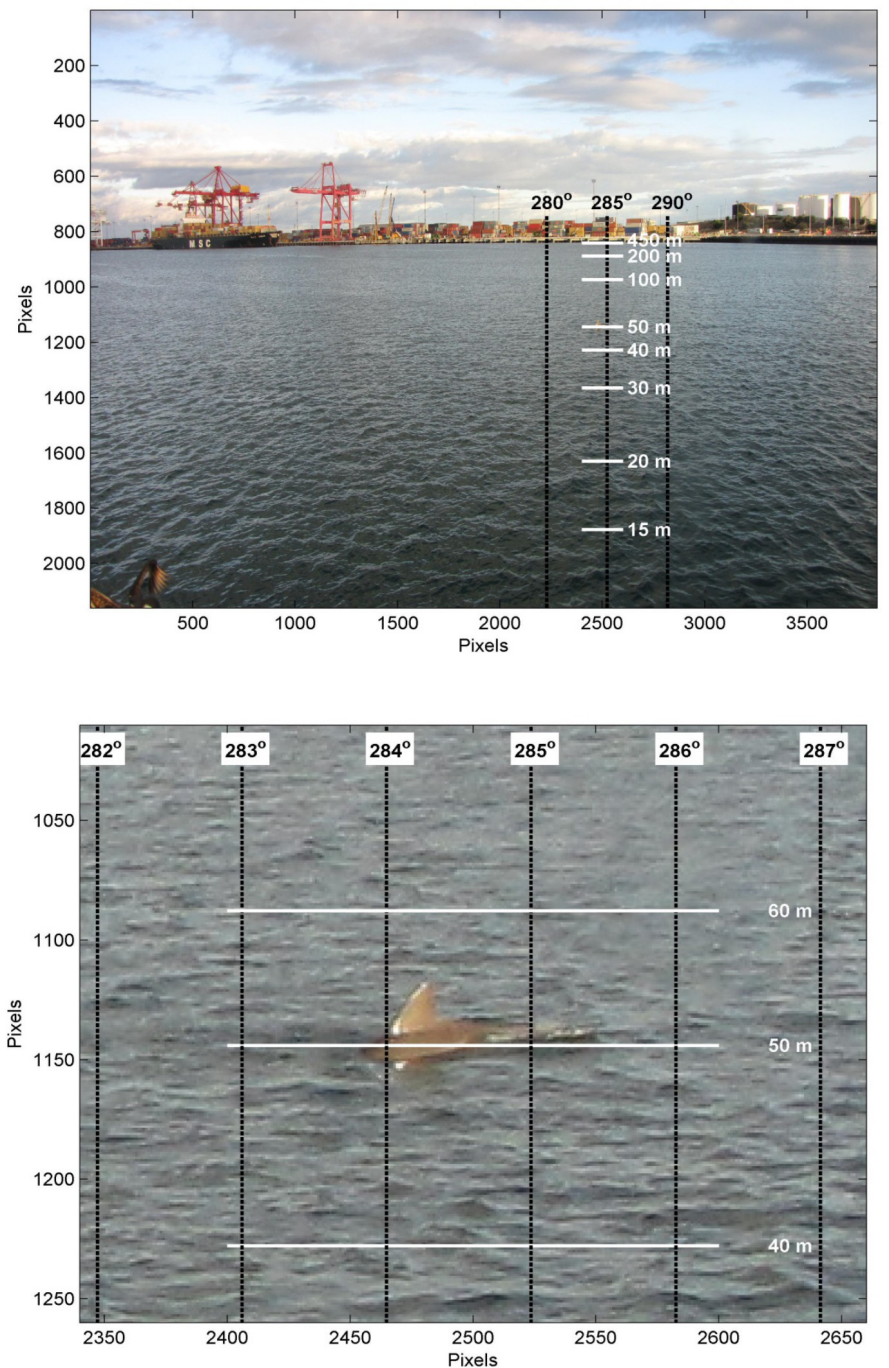
An example image taken with the still camera. Ranges and bearings from the camera (top); zoomed in image to show target (below).

Images from the still and video cameras were used to estimate the position of dolphins at the water’s surface. The positions were calculated by estimating the range and bearing to the dolphin from the camera tower (Fig 3 shows an example image overlayed with calculated ranges in meters and bearings in degrees). For both the video and camera images, the bearing to the dolphin from the camera was calculated by interpolating the bearing from known land marks, such as the flood lights and buildings. The range was based on the angle down from where the water met the wharf on the other side of the harbour, i.e. the wharf was used as an artificial horizon. Firstly, the position of the wharf directly above the dolphin image was determined from ground control points, then the number of degrees down from this position to the dolphin was calculated. Converting the dolphin position in pixels to degrees (relative to the far wharf) could be done using the stored focal length with the images captured by the still camera. The images extracted from the video camera, however, did not store this information. As a result, for the video images the number of pixels to degrees was calculated using the heights of the lights on the opposite wharf that were always in the image. The elevation angle to the dolphin and camera height was then used to calculate the range. The bearing and range to the dolphin from the camera tower could be then used to calculate the dolphin’s position. The geometry calculations required accurate height and position of the cameras, which was carried out using a combination of tape measure and engineering diagrams. The range also required correction for tide, which was done using measurements recorded by a tide gauge in the Fremantle Port Inner Harbour. All of the calculations were carried out in a program developed in Matlab R2011b [33] by one of the authors called ‘Dolphin Image Tracker’.

A calibration test was carried out to assess the accuracy of the method. This involved a boat being photographed and video recorded at different points in the study area. At these points, a red flag was held up so it could be clearly seen in the images and it also served for indicating the time positions were recorded using a handheld GPS on the boat. The positions of the boat taken when the red flag was held up were calculated from the camera and video images and compared to the GPS. This was carried out at nine locations throughout the survey area. The difference between the GPS positions and those estimated using the still camera images ranged between 4 and 29 m, with a mean of 14 m. The difference between the GPS positions and those estimated using the video camera images ranged between 1 and 19 m, with a mean of 12 m. As to be expected there was an increase in error with range, but it was not found to be significant.

### Data analysis

The study area was first described by mapping out the field of view of the cameras. The extent of the area was determined by identifying the coordinates (latitude and longitude) of the left and right-most positions of the field of view where the wharf meets the water at the opposite side of the channel from where the cameras were located. Maps were produced with the aid of ArcMap 10.0 [34].

Detection as a function of range from the cameras were then assessed by plotting all positions of transit events upon first detection. Positions were plotted using algorithms developed in Matlab R2011b [33] described above. Detection as a function of range was then plotted. Comparisons in detection functions between the two cameras were made.

The influence of environmental variables on detectability was also assessed for the video and still cameras. Contributions of each variable to the number of transit events recorded to predicting numbers of transit events recorded was assessed using Generalised Estimation Equations (GEEs). GEEs were produced in R [35] run through RStudio Version 0.98.501–2009–2013 RStudio, Inc., with the aid of three packages, being “doBy” [36], stringr [37] and geepack [38–40]. In this study, the response variable, presence and absence of dolphin group transit events, is measured repeatedly over time at one-hour intervals between 06:00 to 18:00 during 42 days between April and August at the same locations. Applying a generalised linear model (GLM) would violate the independence assumption since there is a longitudinal aspect to the study. Therefore a generalised estimation equations (GEE) was used as a tool to include a dependence structure [41]. An AR-1 correlation structure was selected since it is used for data sets in which there is a time order [41]. In specifying the gee in geepack’s [38–40] geeglm function, correlation structures are created upon specification using the appropriate syntax.

Initially, hours of effort for environmenttal variables at different levels were summarised. Subsamples were taken so that the first observation period for every hour was included in the data analysis for testing. The response variable is the presence and absence of transit events, which was defined by the period from the first detection of a group of dolphins within the camera’s field of view to the time when it was last detected transiting the camera’s field of view. The response variable would be a single value for a group observed surfacing multiple times during its transit across the camera’s field of view. Vectors of all dates and times between the start and end date of field surveys were created to include an autocorrelation structure for observations over time within the model. Observations for every hour were chosen over observations that were more distant temporally (every 4 hours, for example) since the latter would result in significantly reduced subset of data and lower reliability in the interpretation. Rather than “sites” being the grouping structure as in other analyses, here “time block” was considered the grouping structure due to a large gap between the first time block (April 16^th^ – June 2^nd^) and the second time block (June 29^th^ – August 2^nd^) in which the data were collected (hence treated independently).

Autocorrelation tests were carried out. Based on autocorrelation being present and biological knowledge, an Ar-1 auto-correlation structure that allowed for dolphin observations in the Fremantle Port at time “s” to depend on those measured at time “s – 1”, and also, although less strong, on “s – 2”, etc. was used [41]. In preparing the data for analyses, no outliers could be detected. However, the data was reduced to a subset containing the first observation of every hour and observations with corresponding explanatory variables with levels having greater than 20 observations. The final data set had 343 observations after 46 observations were removed as a result of this process". Collinearity (high correlation) between explanatory variables was tested (and one of a collinear pair of explanatory variables removed if collinearity was found), and the relationships between the response variable and the explanatory variables was explored. In order to assess collinearity, pairwise scatterplots were produced, and correlation coefficients and variance inflation factors (VIF) calculated (using the AED package [35]). VIF values below 3 [42] were considered to not be collinear. Binomial distribution was selected (for presence/absence data). The model was restricted a priori to an acceptable level of complexity, based on a general rule of thumb of >20 samples per covariate level [43]. For this reason, only Beaufort and Glare and their interaction were included in the final model as an explanatory variable. Explanatory terms were dropped one by one and each time refit the model, ran validation processes, and compared the model with the previous one using Wald tests to test the significance of nominal variables. Explanatory terms dropped included those of no significance. For model validation, residuals were plotted against each individual explanatory variable to ensure there were no obvious patterns (although interpretation of these for a binomial response variable is limited).

Finally, a cost-benefit analysis was carried out in order to compare the costs of using cameras versus the costs of having trainned observers in the field. Costing parameters used were based on salary scales for general staff from a typical Western Australian University at the time of submitting this manuscript, and expressed in Australian Dollars. The exchange rate between Australian and American Dollars was approximately of equal values (1 USD = 1.07 AUD, average) over the period in which this manuscript was finalised for submission.

## Results

The total sampling effort was 42 days over a period of 3.5 months in 2010, including 9 days in April, 12 in May, 3 in June, 17 in July and 1 in August. Seven hundred and twenty-seven (727) video files totaling 493.4 hours of video footage were analysed during 418 hours. For still images, a total of 3,159 still image files which were taken over 17.6 hours were analysed during 17.5 hours. One or more dolphins were present within the camera’s field of view during 39.8 hours of video recordings, which corresponded to 8.1% of the total period of recordings. For the still images during the 8.1% of corresponding video time, one or more dolphins were present within the camera’s field of view in 346 photos, which corresponded to 11% of the total still image files analysed.

### Area sampled

**Fig 4:**
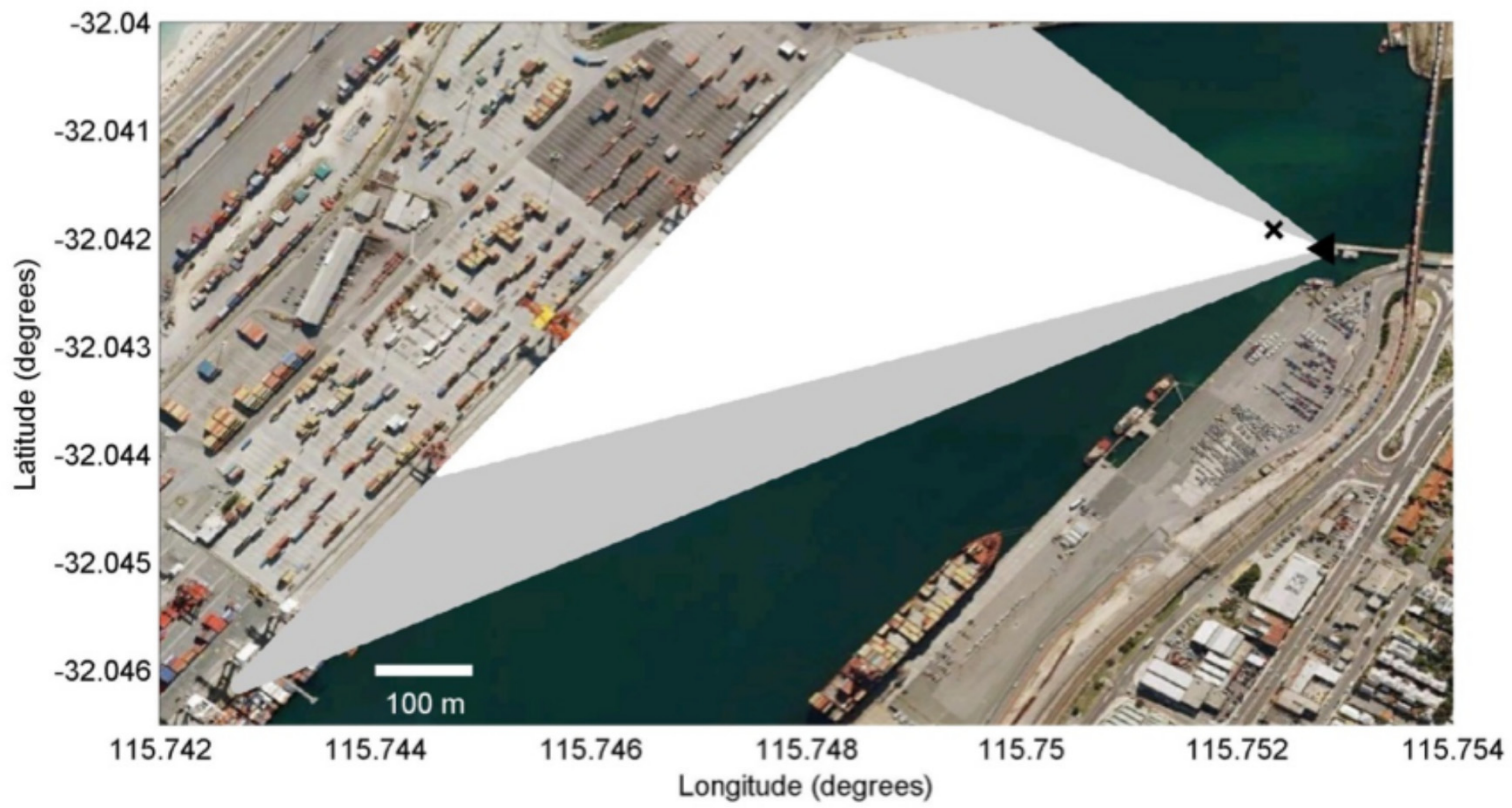
Field of view. The area covered by the still (grey and white) and video (white) cameras and the location of the camera tower (black triangle), and dolphin (black cross).

The area sampled by the video camera comprised approximately 114,000 m^2^ and that sampled by the still camera was 193,000 m^2^, with the largest field of view in the direction of the mouth of the Fremantle Inner Harbour towards the southeast (Fig 4).

### Detection range

**Fig 5:**
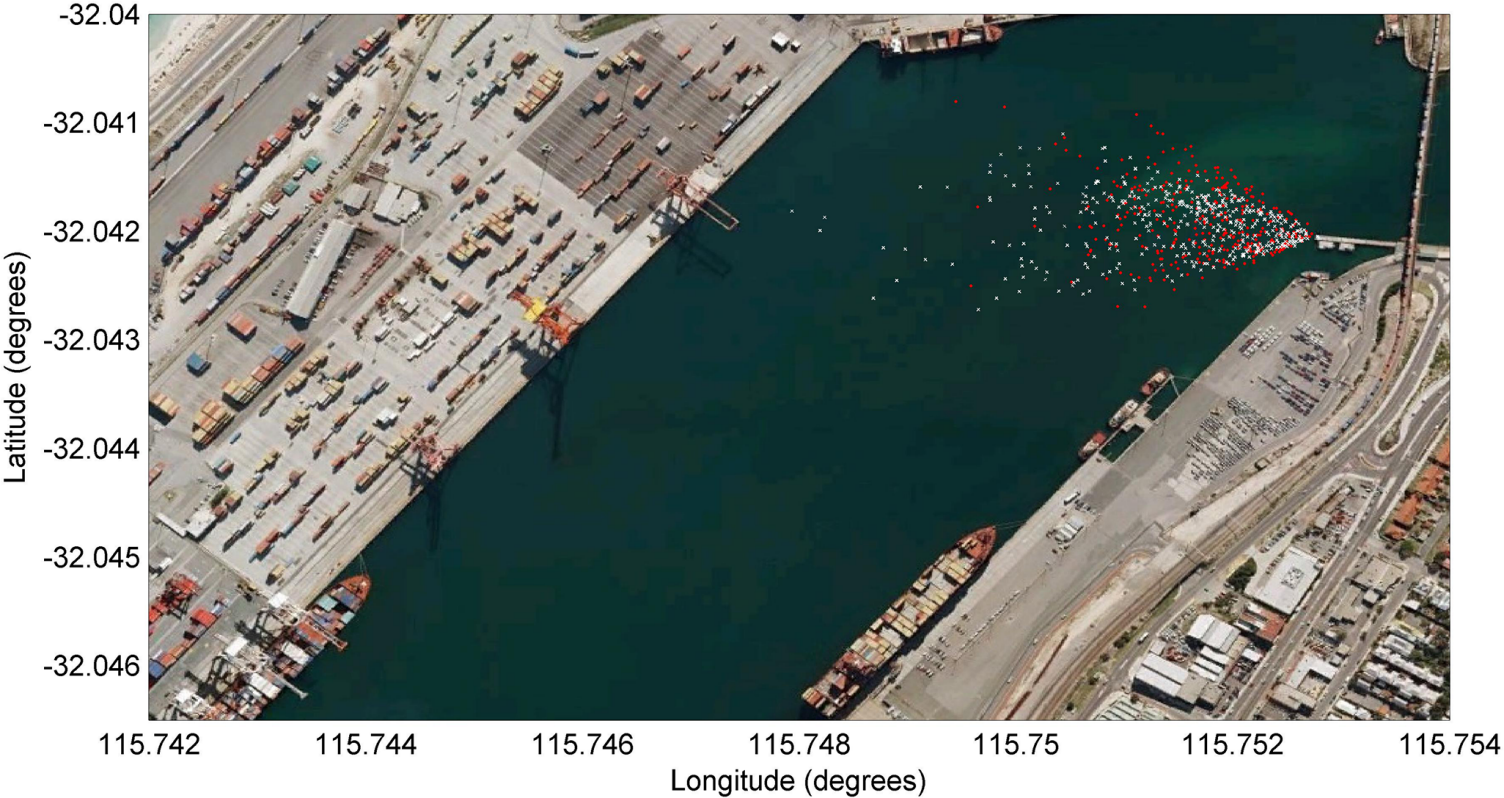
Position of first detected dolphin within a group in the Fremantle Inner Harbour as a function of range and bearing using both cameras. White dots correspond to plots obtained from the still camera and red dots correspond to plots obtained from the video camera.

**Fig 6:**
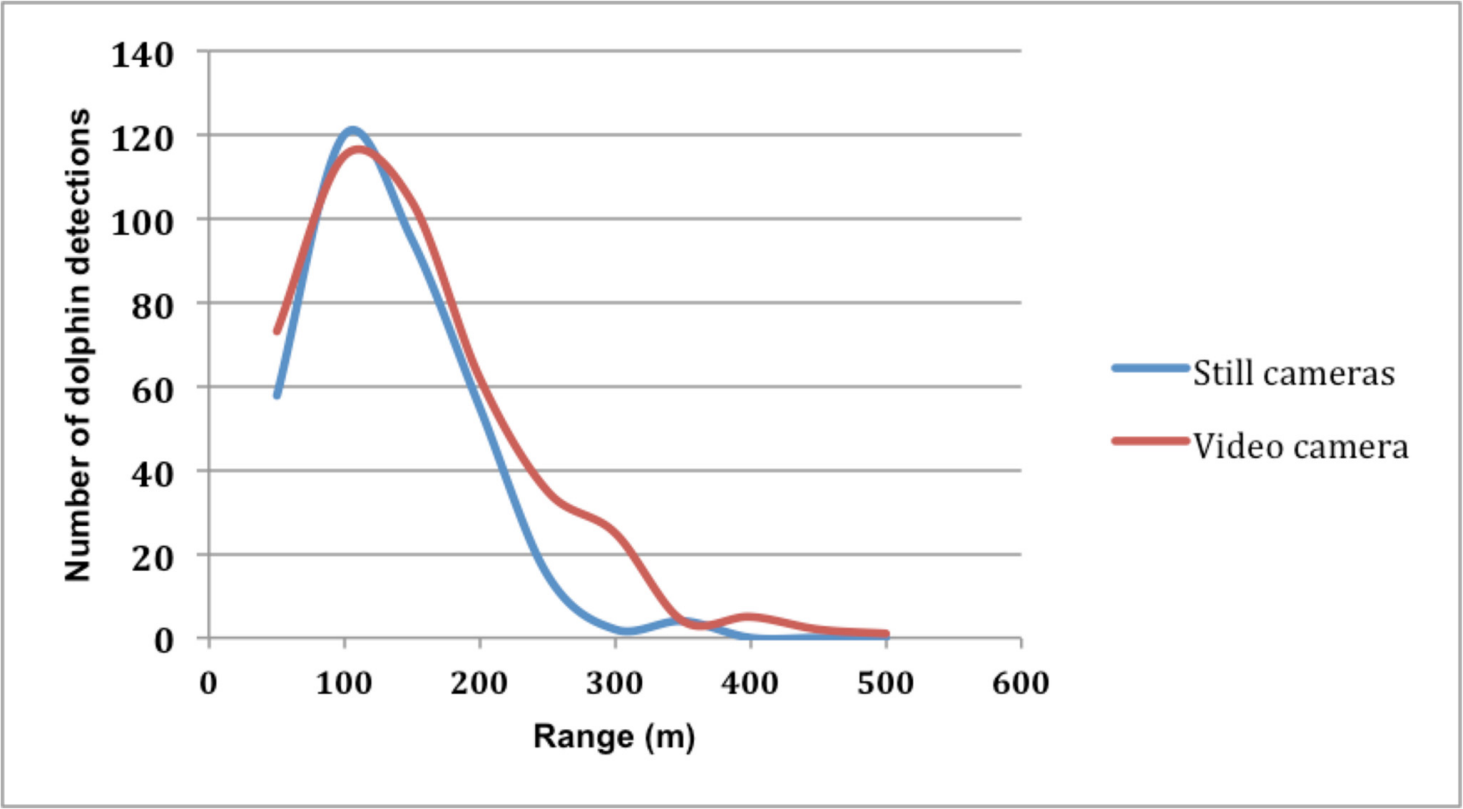
Dolphin detection range. Number of dolphin group detections as a function of range from the cameras (range to the group is based on the position of the first detected dolphin surfacing within the group).

**Fig 7:**
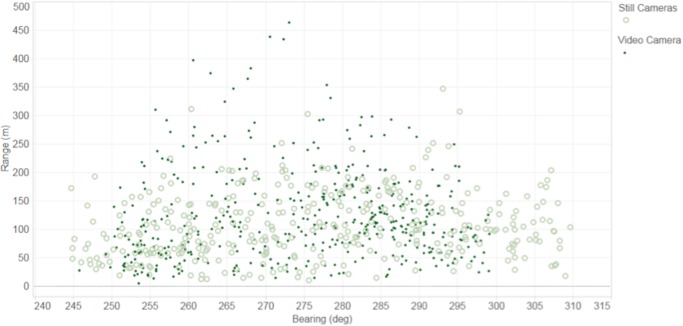
Dolphin detection range versus bearing. Number of dolphin group detections as a function of bearing and range from the cameras (bearing to the group is based on the position of the first detected dolphin surfacing within the group).

Detection of dolphins ranged from 5.6 m (closest detection) to 463.3 m (most distant detection) for the video camera, and from 10.8 m to 347.8 m for the still camera (Fig 5). The number of transit event detections reached a peak at 110 m on both cameras and then gradually decreased within decreasing range to the cameras (Fig 6). For the still camera, the bearing with reference to geographical north varied from 244° to 309°. Similar results were obtained from the video camera, in which dolphins were detected between 245° and 299°. Most dolphins were observed between the bearings of 280° and 285° on the video camera, and between 265° and 270° on the still camera (Fig 7).

### Effect of environmental conditions on detectability

**Fig 8:**
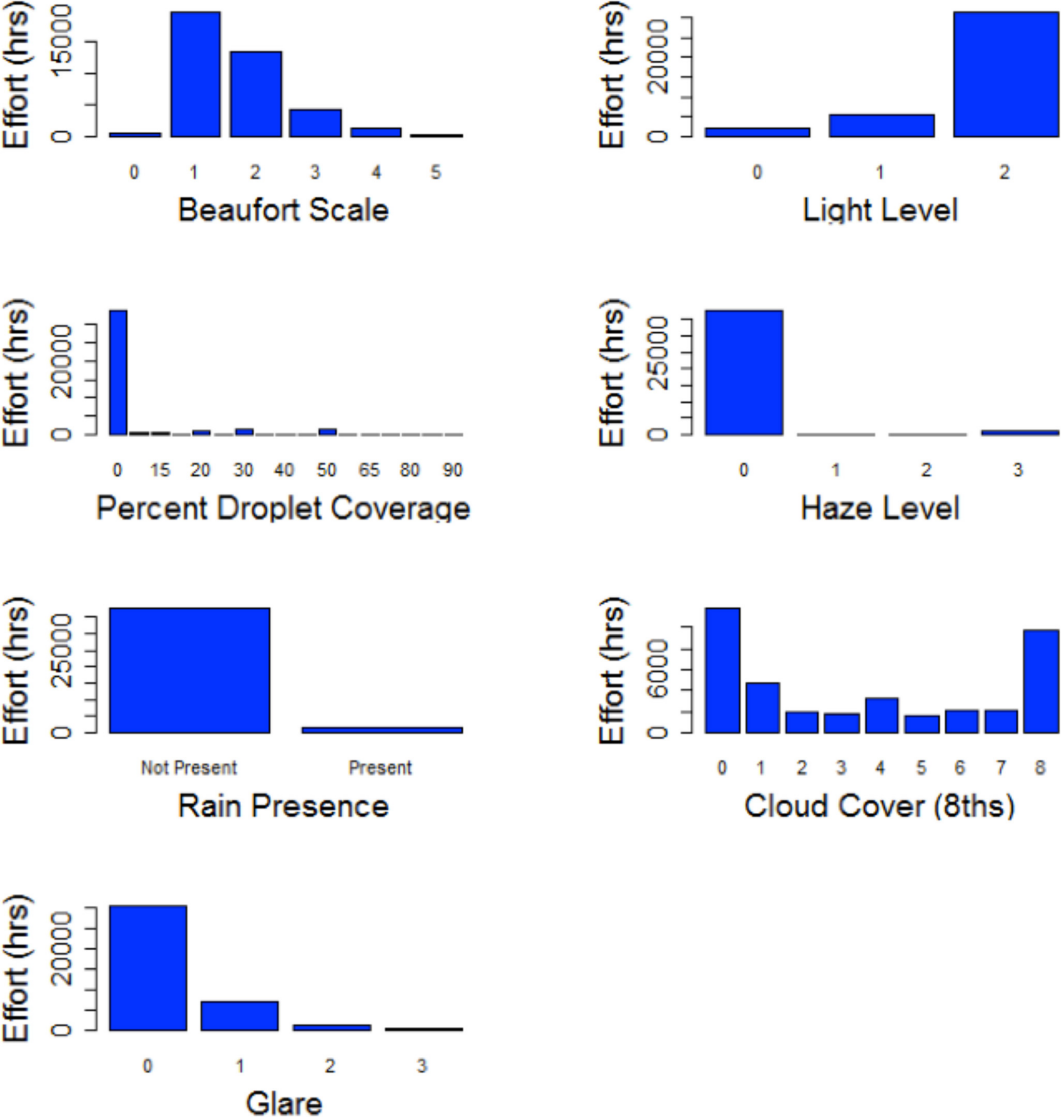
Effort for each environmental variable. Effort (hours) of data collected under each environmental variable (Beaufort Scale, Light Level, Percentage of Droplet Coverage, Haze Level, Rain Presence, Cloud Cover and Glare).

The observations were not significantly affected by haze or rain (rain obscuring the view and droplets on the lense window) as these occurred rarely (Fig 8). Percentage of droplet coverage, haziness, and rain were removed from the analysis because they did not have enough information in most levels. Most data were collected during light levels of 2, for the reason that there were more hours between 06:00 and 18:00 with light (Fig 8). The majority of cloud cover conditions were either no cloud cover or 100% (scale of 8, Fig 8). Cloud cover was correlated with Beaufort, however, so Beaufort was selected as the most important of the two variables to include in the model.

The majority of data were collected during glare conditions of 0, and to a lesser extent 1. Glare never covered the entire field of view of the cameras so glare levels of 4 were never recorded. Data collected during glare levels of 2 and 3 were excluded since the sample size after subsetting the data to include the first observation periods of every hour was not large enough to include in the model.

The effect of environmental glare and Beaufort (which had sufficient samples across the measured levels) on detectability was assessed by finding the best fit GEE. Glare only included levels of 0 and 1 out of the scale of 0 to 4 since the number of observations were too few in glare scales 2 to 4 to include. Also, Beaufort scales 0, 4 and 5 were excluded from the model due to their small sample size. The model including glare only as an explanatory variable was the best model. Beaufort and its interaction with glare were not significant. The Wald test showed no difference between the submodel composed of glare, Beaufort, and their interactions as explanatory variables and the submodel with only glare and Beaufort included. Glare remained significant in all models.

The final, best model was given by

ηis=0.246+−0.177×Glare

with time block given by i and the time (over the study period) in the autorrelation structure given by s (Table 1). The correlation of dolphins being observed between two sequential times was 0.657, which is relatively high. Its standard error (0.036) was small, indicating that the correlation was significant. The estimated correlation parameter indicated the presence of auto-correlation within periods of observations, justifying the decision of using GEE. The resulting interpretation for the analysis was that glare was associated with a decrease in dolphin transit detections (based on the negative sign on the parameter estimate of Glare, -0.177).

**Table 1:** Generalised Estimating Equations model to identify significant predictive variables in detecting transit events.

Parameter Coefficients	Estimate	Std Err	Wald	Pr(>|W|)
(Intercept)	0.262	0.118	4.89	0.027 *
Glare	-0.264	0.113	5.41	0.020 *
**Estimated Scale Parameters** (Intercept)	1.04	0.0244	-	-
**Estimated Correlation Parameters** (alpha)	0.385	0.0455	-	-

Significance code: 0.01 = ‘*’.

**Fig 9:**
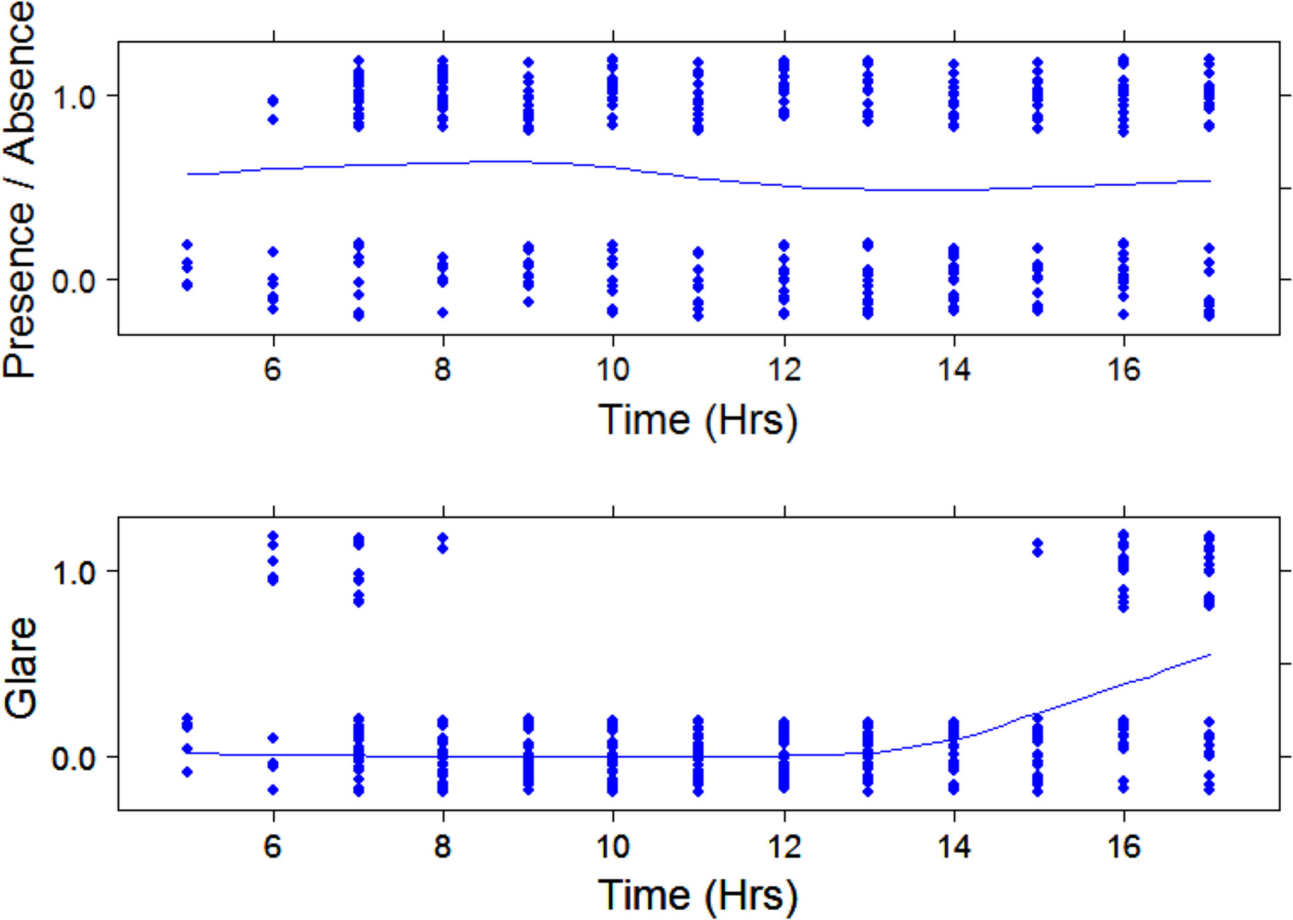
Presence/absence of dolphins over time (top) and levels of glare (bottom) over time. Values on y axis are “jittered” to facilitate displaying of overlapping observations.

To ensure that dolphin transit events (presence/absence) were not influenced by a coincidence of transits having a pattern of occurring in the Fremantle Port area when glare was lower (even though auto-correlation over time was included in the model), dolphin observations and glare were plotted over time and a submodel was produced which only included data during hours when glare could either be present or absent (due to clouds was included). Glare of 0 (no glare) occurred always during the middle of the day from 09:00 to 15:00, however dolphin transit events occurred throughout the day, (Fig 9). The submodel which excluded the hours between 09:00 and 13:00 did not differ in glare being the only significant explanatory variable.

### Cost effectiveness of using cameras

The cost effectiveness analysis consisted of a comparison of the total cost of the collection of the data, including expenses of equipment, labour for data collection, equipment maintenance and photogrammetry programming (Table 2). The cost of overheads, on-costs (a cost that the employer incurs, such as the usual benefits, when employing someone in addition to the salary or wages), administrative support, and statistical analyses were not included. For the camera method, costs included gear, labour for gear preparation, field servicing, photogrammetry programming and video perusing. For the estimate of the more traditional observational methods undertaken by field personnel, costs included gear and labour related to data collection. Labour of field personnel was based on a minimum of two people required on site 13 hours per day (one operating a theodolite for positional information, and the other spotting with binoculars and inputting all data in a spreadsheet in a computer). Although normally field staff would not work during poor weather conditions, most days in which cameras-based data were collected for this study was during reasonable weather conditions for field work. The additional hour per day (ontop of the 12 hours of observations) was included for general tasks such as equipment cleaning. The gear would consist of a pair of binoculars, a theodolite, a computer and batteries. The total estimated cost and time spent for the camera method was USD$30,485 and 490 hours (including all labour costs), and for the field-based observations, USD$47,580 and 588 hours. The largest difference in cost between the methods was in labour, which was total of USD$24,265 for the camera method and USD$36,280 for the field-based observations.

**Table 2:** Costs for camera system and personnel in the field (in US Dollars).

Item	Cost ($)
**Camera system**	
Gear	5,014
Labour gear preparation	5,900
Labour field servicing	3,471
Labour photogrammetry programming	2,647
Labour video perusing	12,247
Expendables (batteries, hard drives, SD cards)	1,206
Total	30,485
**Personnel in the field**	
Gear	10, 359
Labour	36,280
Expendables	941
Total	47,580

## Discussion

Apart from the high initial cost with camera equipment and set up, the cost effectiveness analysis showed this methodology is financialy advantageous when compared with traditional field work for the same purposes. The fact that training is required to use a theodolite (trained theodolite observers for tracking dolphins are not common) makes it less attractive as specific knowledge is involved and therefore the salary scale is higher. Furthermore, man-hours required would be fewer using the camera method compared to the hours required for field-based data collection due to the possibility of watching the videos at a speed 1.75 times faster than normal speed. More than 493 hours of video footage were analysed in 418 hours, including time taken for rewinding, pausing and watching the videos at normal speed to check for details on dolphins, which is a significant advantage because it increases the reliability of data collected. A period of 12 hours of video footage could be analysed in as short of a period as 7-hours if dolphins are not present or if they are not very active. The camera method would take 84% of the time that it would were it obtained by personnel in the field. Furthmore, at locations and seasons in which weather conditions are poor, sample size from data collected in the field could be significantly limited.

The camera method also allows for the images to be stored and accessed for post reference if needed. Camera trap images support the collection of additional details about target species, such as individual identification and activity patterns [7]. When using theodolites for cetacean surveys, there is a larger risk of missing the target or an event than when using cameras since the target is still in the image and the videos can be rewinded and paused. Also, during field work, data collection is more subject to obsever bias as the event usually occurs very quickly and is registered at the time it happens, not allowing time for double checking. The advantage that observers in the field have is having a wider field of view and because of this being able to “follow” individuals for longer periods of time in the study area.

Aside from the differences in costs and trade-offs using the two methods, there is also a difference in the implications for health and safety. There is an increased health and safety risk associated with having a team of observers in the field for long periods of time. For example, there is an increased risk of fatigue, heatstroke, heat exhaustion, hyperthermia, or hypothermia. In remote areas, there can be other risks such as increased chances of snakeites, or falls in rocky or uneven terrain due to more frequent visitations to the site. The salary scale here considered for comparisons related to costs for data collection was based on a casual research assistant with basic experience in observation of marine mammals. If personnel with more knowledge and field experience were required, the cost would be considerably higher. The cost effectiveness analysis did not consider the costs regarding safety equipments such as hat, boots and sunscreen.

The range at which dolphin groups were detected of up to approximately half a kilometer from where the cameras were located showed that cameras were able to detect dolphins within a relatively long range when compared to results that displayed dolphin detectability of up 300 m in areas investigated through standard line-transect survey methods [44]. The use of higher quality cameras in the current study could mean an improvement in detection range, however there would be the drawback in that the cost would increase. Furthermore, the improvement in detection capability of more expensive cameras would need to be ascertained whether they significantly improve the outcome of studies using them. Dolphins have been seen using the entire study area regularly (this is currently being quantified and has not been published yet). It is likely that field observers positioned at the location where the camera tower was placed using binoculars to aid detection could detect dolphins at greater ranges, however from the authors’ experience at this site, it would likely not be beyond 200 or 300 meters more. Furthermore, considering observers would not be at the same altitude above sea levels as the cameras, using a theodolite at this low level would introduce a significant error in positional information due to the small change in vertical angle from the horizontal plane of the theodolite down to the dolphins. For the dolphin detections, a decline in groups detected was expected as the range between dolphins and cameras increased. This was true, except for within ranges close to the cameras, where detections declined from their peak within the interval of 100 m to 110 m from the cameras. This is likely a function of the relatively small area being sampled close to the camera. In the present study, it is important to consider that the dolphin positions recorded were documented when dolphins first appeared in the field of view in order to obtain unbiased results. If dolphins have already been detected within the video, for example, and direction of travel and behavior have been noted, then the observer has a greater chance of detecting any subsequent surfacings even if they are at larger distances from the camera than a first detection might allow.

The area covered by the still camera was slightly larger than that covered by the video camera, which was reflected in the difference between the maximum and minimum bearings in which dolphins were observed (65° for the still camera and 54° for the video camera). However, the larger field of view of the still camera did not overcome the main limitation in using cameras which is the limited field of view [23, 24]. The camera system does not provide a wide field of view, which is of particular interest in studies which require individual identification, tracking of groups and subgroups, and/or group composition. Also, improved detection of dolphins is likely from traditional field-based visual methods. In this study, dolphin detections were recorded as transit events in the video images due the difficulty in identifying dolphins individually, which would have only been possible when they were very close to the camera.

The still camera was more limited than the video camera in that the probability of dolphins being on the surface of the water when the still images were taken reduced the number of detections significanctly. If a species occurred rarely in an area, a video camera should be used over a still camera. If still cameras are to be used, a still camera pair could be useful to obtain information on positions of animals in areas where there are no reference points available. Also, in remote areas, solar panels (rather than car batteries which were used in this study) would advantageous since they require little servicing.

While cameras can operate for long periods under a variety of weather conditions in water tight boxes, including drizzle and rain, variation in environmental conditions influenced detectability of dolphins. In this study, rain and haze did not occur often, so did not affect detectability. However, at locations where there are significant poor weather conditions, their effects should be considered before implementing cameras for detecting animals since images and videos taken during heavy rainy periods or during high wind speeds were often partially obscured by water droplets on the outer windows of the water-tight box. Having said this, the camera system was still advantageous in that information was collected even in poor weather conditions when a field team would not operate. Dust on the camera lens combined with the reflection of the sun also obscured the view of the camera on a number of occasions, hence cleaning the lenses periodically is important. Also, this work was conducted in a relatively dry climate. It is unknown whether in humid conditions the windows of the encasing protecting the cameras would be fog up with condensation.

In terms of glare and Beaufort, moderate levels were considered in statistical analyses. Extreme glare and Beaufort 4 or above were not considered in statistical analyses here due to their relatively low sample size, but are expected to have an affect on detections using cameras as they due for observations made directly in the field. Therefore the low sample size did not allow to investigate the extent of the effects of these variables on detectability at higher glare levels and sea states. For a camera heigh of 20 m, however, waves and swell with a height of 0.5 m may not represent a significant bias, mainly if the target is present in the field of view for prolonged periods [25].

At more moderate levels, detections were effected by glare. Potential masking effects caused by sunlight were also observed in a study using cameras mounted on an unmanned vehicle when analyzing images of dugongs taken during an aerial survey [45]. The described effect however was not due to glare, rather it was due to grouping of sun gleams reflecting off ripples [45]. Despite the drawback of long post-processing time due to assessing the quality of the data collected, the use of remote video cameras can facilitate studies such as predator-prey relations, allowing investigations on predatory patterns of killer whales [46].

Given that the most significant cost was labour in perusing the camera images, the authors suggest that the development and testing of an algorithm for automatic or semi-automatic dolphin dection would be invaluable. However, extensive testing and refining the algorithm would be required since there are constant changes in the background colour of the images. The amount of light reflected on dolphins and their surface behavior change their appearance. Furthermore, there were occasions during which all that was seen was a splash and/or a point of a fin, which could be misdetected due to similarities in the way the cues look to splashes from diving birds such as seagulls or birds in the distance. The regular boat traffic captured by the cameras and the constant changes in dolphins’ orientations would represent additional aspects to be considered when developing an effective automated dolphin detection algorithm. The algorithm written for dolphin positioning, however, proved to be of great benefit. The algorithm effectively allowed relatively accurate calculation of dolphin positions from video grabs and still images, dispensing the use of a theodolite.

Overall, camera systems can be of great advantage for the collection of long-term data of activity and occurrence of animals in an area, and can be especially useful in remote areas if the system is fitted with solar panels and a remote data acquisition system. They also have the advantage of operating continuously regardless of weather conditions and still provide useful data. Furthermore, the use of cameras to detect dolphins is more cost effective than having personnel in the field, and reduces the health and safety risks to field staff. This study showed that cameras could be effectively implemented onshore for research such as studying changes in habitat use in response to development and construction activities.

## Conclusions

This study identified high definition video camera as a valuable tool to study bottlenose dolphins in the wild over long periods based on detection performance ability to obtain relatively accurate positions of dolphins. The limited field of view was the main disadvantage of the methodology, and this should be carefully considered prior to usingcameras for data collection before undertaking a research program. Cameras could be used to study abundance of marine mammals if reliable assumptions can be made about the target study animals such as frequency of individuals transiting the field of view of the camera and their occupancy of the study area. Environmental conditions (particularly presence of glare) did show an effect on detectability, but this bias would likely also exist in data collected by observers in the field. The camera system is particulary advantageous over personel in the field in poor weather conditions when field work could not be undertaken and for studies requiring acquisition or perusal of observations a repeated number of times since video footage and images can be stored. Finally, the time saved by watching video footage at faster speeds result in a more cost-effective and practical approach than field observations for long-term studies, and can be used to address unanswered questions for improved management and conservation of cetaceans.
